# Grapheme-Color Synesthesia and enhanced Working Memory for the materials that induce synesthetic experiences

**DOI:** 10.1192/j.eurpsy.2023.1940

**Published:** 2023-07-19

**Authors:** M. Ayobi, E. Molchanova

**Affiliations:** 1 Psychology, American University of Central Asia, Bishkek, Kyrgyzstan

## Abstract

**Introduction:**

This study investigated the influence of synesthetic experiences on working memory and hypothesized that Grapheme-Colour Synaesthesia causes enhanced working memory for the materials in the congruent condition.

**Objectives:**

The current study uses the existing experiments conducted within the field of Synaesthesia as a basis in order to find out whether synaesthesia influences working memory for the letters, words, and days of the week that elicit synesthetic experiences

**Methods:**

Experimental research design was used to identify the extent of the causal relationship between Synaesthetic and non-Synesthetic experiences and enhanced working memory in both Synesthetes and non-Synesthetes. A short screening questionnaire, Stroop task, and N-back task was used to measure the relationship between the two variables for quantitative measurement. (Radvansky, 2011), This study uses materials such as Stroop task and n-back task from Robinson’s (2015) work, Radvansky’s (2011), and Terhune et al. (2013) work. However, certain changes in the methodology of the current study makes it easier and efficient to conduct this study with a different population.

**Results:**

Grapheme-Color synesthetes (Mean= 1276.682 milliseconds) appear to take less amount of time in responding correctly to the incongruent stimulus in the Stroop Task than non-Synesthetes (Mean= 1487.89 milliseconds). Secondly, Grapheme-Color Synesthetes (Mean=1170.929 milliseconds) have a significant difference in accuracy of responding in the congruent condition of the Stroop Task with the non-Synesthetes (Mean= 1491.159 milliseconds). Further evidence from the N-back Task also demonstrated a significant relationship between the variables in both incongruent and congruent conditions; Grapheme-Color Synesthetes (Mean= 2621.390 milliseconds) showed a significant difference in correctly responding to the nonmatching stimulus in N-back Task with Non-Synesthetes (Mean= 2854.351 milliseconds). Similarly, Grapheme-Color Synesthetes (Mean=1330.130 milliseconds) showed a large difference in responding correctly to the matching stimulus in the N-back Task with Non-Synesthetes (Mean= 2301.071 milliseconds). Grapheme-Color Synesthetes were faster in responding correctly than non-Synesthetes in all conditions.

**Conclusions:**

Regardless of what have been concluded as a result of the current study, it is difficult to reach at any conclusions taking into account the oppositions about working memory of Grapheme-Color Synesthetes in different studies. Therefore, the data suggest that future studies should further test the capacity of working memory in Grapheme-Color Synesthetes.

**Disclosure of Interest:**

None Declared

